# Evaluation of Chloride-Ion Diffusion Characteristics of Wave Power Marine Concrete Structures

**DOI:** 10.3390/ma14195675

**Published:** 2021-09-29

**Authors:** Changhyuck Lim, Gyuyong Kim, Gyeongtae Kim, Bokyeong Lee, Youngduck Kim, Seungho Shin, Jeongsoo Nam

**Affiliations:** 1 Marine Renewable Energy Research Division, Korea Research Institute of Ships and Ocean Engineering, 32, Yuseong-daero 1312beon-gil, Yuseong-gu, Daejeon 34103, Korea; ckdgur1092@kriso.re.kr (C.L.); kyd000@kriso.re.kr (Y.K.); shinsh@kriso.re.kr (S.S.); 2 Department of Architectural Engineering, Chungnam National University, 99, Daehak-ro, Yuseong-gu, Daejeon 34134, Korea; j.nam@cnu.ac.kr; 3 Technical Support Team, Halla Cement, 70, Iseopdaecheon-ro 561beon-gil, Hobeop-myeon, Icheon-si 16078, Korea; gyeongtae.kim@hallacement.co.kr; 4 Construction Test and Assessment Center, Korea Institute of Civil Engineering and Building Technology, 283, Goyang-daero, Ilsanseo-gu, Goyang-si 10223, Korea; bokyeonglee@kict.re.kr

**Keywords:** marine concrete, hydrostatic pressure, chloride-ion penetration, capillary pore, wave power

## Abstract

Wave power marine concrete structures generate electrical energy using waves. They are exposed to a multi-deterioration environment because of air and hydrostatic pressure and chloride attack. In this study, the effect of air pressure repeatedly generated by water level change of wave power marine concrete structures on the chloride-ion diffusion of marine concrete was analyzed. The chloride-ion diffusion of wave power marine concrete structures was evaluated. The results show that the air chamber and bypass room, which were subjected to repetitive air pressures caused by water level changes, showed a higher water-soluble chloride-ion content compared to the generator room and docking facility, which were subjected to atmospheric pressure. Field exposure tests and indoor chloride attack tests were performed using fabricated specimens to analyze the effect of pressure on chloride-ion penetration. It was confirmed that Portland blast furnace slag had a greater inhibitory effect on chloride-ion penetration than ordinary Portland cement. The concrete specimens subjected to pressure showed increased capillary pores and micro-cracks. We devised an equation for calculating the diffusion coefficient based on measured data and estimating the diffusion coefficient for the location receiving repeated air pressure by using the diffusion coefficient of the location receiving general atmospheric pressure.

## 1. Introduction

Oceans constitute approximately 75% of the Earth’s surface, and they consist of significant energy resources of various types. Marine energy is a clean energy source that can overcome the problems of environmental pollution and resource depletion due to the use of fossil fuels. Wave energy conversion modules generate renewable energy by producing electrical energy using waves in the sea. Among various wave energy conversion modules, oscillating water columns (OWCs) convert air flow into turbine rotations, where air flow is generated by the upward and downward movements of water columns in structures created by waves. The technology readiness level of OWCs is 6–8, and empirical research on OWCs in actual seas is being conducted worldwide [1,2,3,4,5].

In South Korea, the first offshore OWCs were constructed on the west coast of Jeju Island in 2016 [6]. Most OWCs are fabricated from concrete so that they can be used as breakwaters. They have low durability owing to the following reasons: stress on concrete caused by sea waves, accelerated corrosion of steel due to dry and wet conditions, physical erosion due to the freezing expansion of water in concrete, chloride attack caused by the corrosion and expansion of reinforcing bars due to the diffusion of salts such as sulfates, and carbonation due to low concrete pH caused by carbon dioxide in the air. Chloride attack is the principal cause of deterioration because it decreases the strength of structures and leads to cracks owing to the corrosion of reinforcing bars [7,8,9,10,11,12].

Reinforcing bars corrode when calcium chloride and chlorine ions contained in seawater penetrate the concrete surface and reach the reinforcing bars through potential difference or diffusion. The chlorine ions that reach the reinforcing bars generate a potential difference between the healthy and defective parts; they act as an electromotive force to form a battery that causes corrosion by moving electrons through the reinforcing bar. At the anode, iron is ionized and deteriorates through oxidation according to the generation of electrons, and at the cathode, reduction occurs owing to the consumption of electrons to generate hydroxide ions. As a result, hydroxide ions move to the anode and remain as black rust with insufficient oxidation to form a layer of rust on the iron surface [13,14,15,16,17,18,19].

When free chloride ions reach a concentration that causes corrosion in reinforcing bars, the protective film, which is referred to as the passive film, is destroyed, and the cross section of the reinforcing bars decreases. Thus, the overall strength of the structure decreases.

Concrete is a material through which liquid substances can permeate, and when concrete is exposed to an environment with significant amounts of chloride, chloride permeates through capillary pores, cracks, and defects in the concrete. The mechanism of chloride movement through concrete is largely divided into diffusion, a phenomenon in which chloride moves due to a difference in chloride concentration, permeation, where chloride moves due to a pressure difference inside the concrete, and electrical migration, where chloride moves due to a potential difference. The movement can be further categorized as moving convection, where chloride enters the concrete owing to the difference in water content between the concrete and the atmosphere. 

In general, diffusion theory is based on Adolph Eugen Fick’s mathematical model. The application of Fick′s law to the analysis of chloride diffusion properties in concrete was initiated by Collepardi, and since then, various test methods and analysis techniques have been proposed by numerous researchers to analytically evaluate chloride diffusion properties. Fick′s first law holds in the steady state and states that diffusion increases as the concentration difference and distance increase. Fick′s second law is an application of the first law, which deals with diffusion in an abnormal state and expresses concentration diffusion as a relationship between time and distance. The main mechanisms of chloride migration in marine concrete structures in this study are diffusion and penetration through chloride concentration and air pressure, respectively [20,21,22,23,24].

OWCs consist of an air chamber and a bypass room, where positive and negative pressures are alternately generated by water level changes. These pressure changes can reduce the durability of OWCs by accelerating the penetration of seawater into concrete microstructures [25].

Therefore, in this study, the chloride penetration durability of marine concrete structures was evaluated by measuring the chloride-ion penetration depth and chloride-ion content at locations with low durability, such as the outer walls, air chamber, and bypass room. In addition, chloride-ion penetration behaviors were evaluated using an indoor chloride evaluation device to consider the effects of air chamber conditions under positive and negative pressures. In general, many studies have been published on the durability of marine concrete exposed to the marine environment or caused by sea water pressure [26,27,28,29]. However, in this study, chloride-ion diffusion due to air pressure in marine concrete structures was analyzed for changes in the microstructure and pore structure of the concrete matrix.

In addition, the diffusion coefficient that represents the rate of diffusion was calculated using Fick′s second law, which is the diffusion equation, based on the depth measurement data of the OWC offshore concrete structure. In this study, we propose an expression that can estimate the diffusion coefficient of an environment subjected to repeated air pressure (bypass room) using the diffusion coefficient of the marine concrete structure docking facility subjected to general atmospheric pressure.

## 2. Overview of Wave Power Marine Concrete Structure

### 2.1. Wave Power Marine Concrete Structure in Jeju Island

**Figure 1:**
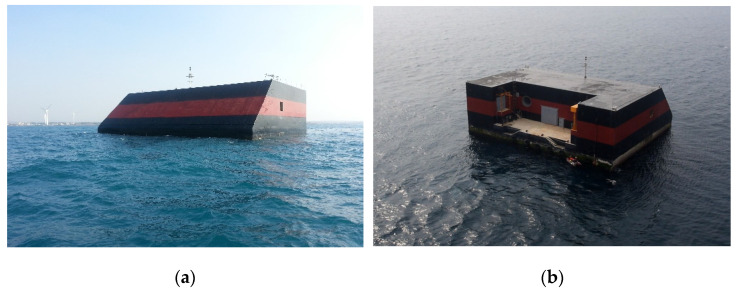
Oscillating water column wave energy structures (OWC): (**a**) Front (air chamber); (**b**) Rear (docking facility).

**Figure 2:**
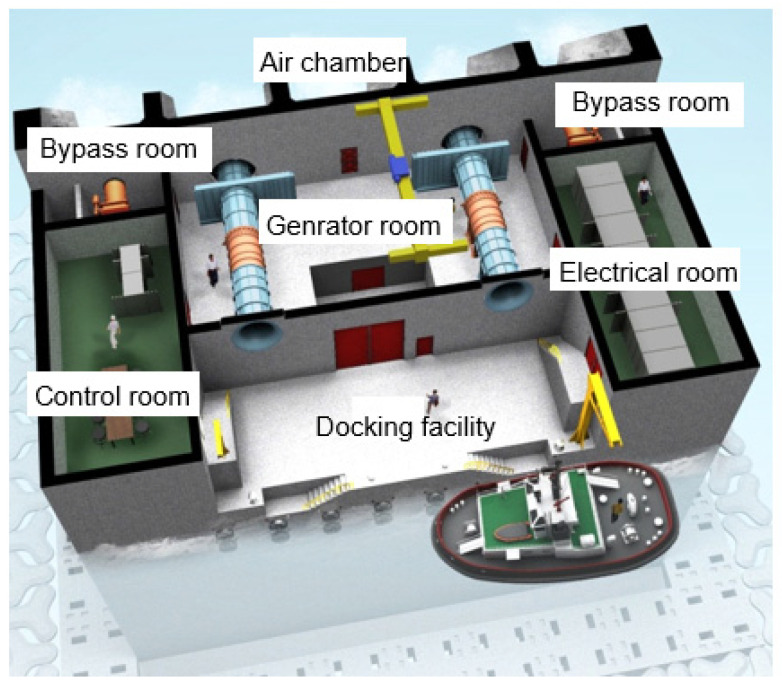
Composition of OWC.

In South Korea, a wave power marine concrete structure is located off the coast of Chagwido Island to the west of Jeju Island. It is a submersible structure fabricated from reinforced concrete caissons of depth of water approximately 16 m. The weight of the structure is 14,650 tons, and its dimensions are 37.0 (L), 31.2 (B), and 27.5 m (H). As shown in Figure 1, the structure is approximately 1.2 km away from the coast, and it is connected to an onshore control room on the beach via submarine cables, which transmit electrical energy. This structure was constructed by replacing 40% of the cement with blast furnace slag cement, and the design compressive strength was 24 MPa. As shown in Figure 2, it consists of a chamber, a generator room, in which an energy conversion module is installed, and a control room, in which a power conversion system is installed. The air chamber and bypass room are exposed to an environment that can cause deterioration owing to pressure changes and chloride attack, and they are subjected to repetitive air pressures of up to 2–3 atm.

### 2.2. Principle of OWC and Causes of Concrete Deterioration by Marine Environment

**Figure 3:**
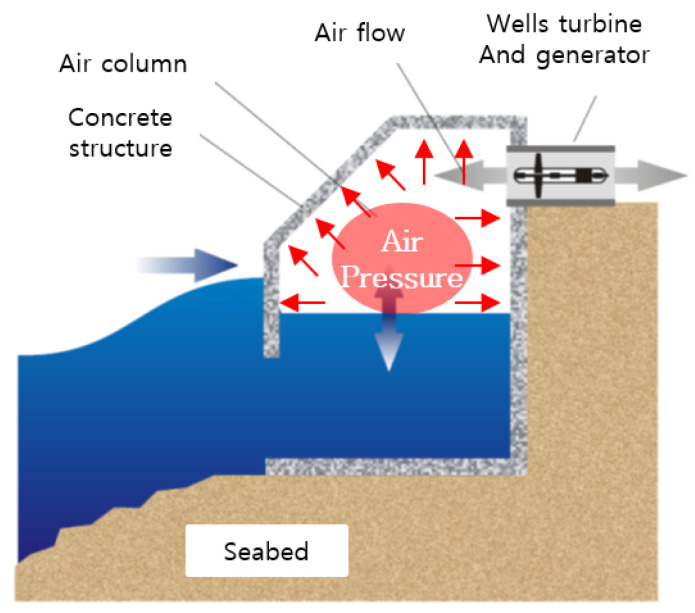
Pressure change in air chamber of OWC.

In wave power conversion, a turbine is driven by converting the kinetic and potential energies of waves to the motion of machines to produce electricity. This technology is suitable for sea areas with large wave heights and extended wave periods. On the basis of the energy conversion principle, wave power converters are classified into the oscillating body, OWC, and overtopping types. An OWC generates electricity by converting wave energy into air flow and placing a turbine in the generated air flow, as shown in Figure 3. Standing waves are formed when incident waves are reflected from the front of the OWC, and the resulting upward and downward movements of the water surface are transmitted inside the air chamber through an opening in the OWC. As a result, the air inside the air chamber undergoes repeated compression and expansion, and this creates air flow in the duct at the top of the air chamber. This air flow drives the turbine and activates a generator, thus producing electrical energy [30].

The repetitive upward and downward movements of the water surface generate air pressure in the air chamber and in the bypass room installed to control the air flow rate. The repetitive air pressure changes and high humidity of the air chamber and bypass room can rapidly degrade the durability of the concrete structure by accelerating the penetration of seawater into concrete microstructures.

## 3. Experimental Design and Method

### 3.1. Overview of Experiment

Experiments were performed to evaluate chloride-ion penetration behavior. Samples were collected from an OWC in an actual sea area to evaluate the chloride penetration durability of wave power marine concrete structures. In addition, indoor chloride attack and field exposure were evaluated using fabricated specimens in an actual sea area to evaluate the effect of pressure on chloride-ion penetration. The design of experiments is shown in Table 1.

### 3.2. Evaluation of Chloride-Ion Penetration Behavior Using Samples from OWC

#### 3.2.1. Design of Experiments

**Figure 4:**
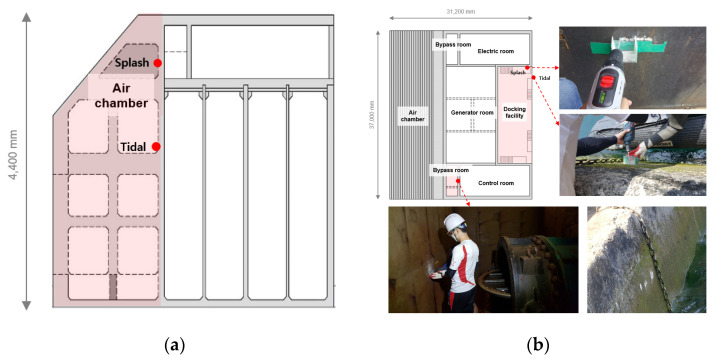
Sampling locations and images of OWC: (**a**) air chamber; (**b**) bypass room, docking facility.

The OWC was divided into the air chamber, bypass room, generator room, docking facility, tidal zone, and splash zone, and samples of pulverized concrete were collected from each location. The water-soluble chloride-ion content was analyzed at different depths to analyze the chloride-ion penetration trends at the locations that were subjected to pressure and the locations that were not. Figure 4 shows the sampling locations and images of the OWC. Samples were collected at depths of 0, 5, 10, 15, 20, and 25 mm. The detailed design of experiments is presented in Table 2.

#### 3.2.2. Experimental Method

The collected samples were filtered through a 0.85 mm mesh, and 3 g of the samples was collected. Then, the samples were measured using a potentiometric titrator (AT-500 N) according to KS F 2713 [31]. The chloride-ion content in the samples was determined by measuring water-soluble chloride ions. The potential of a selective ion electrode was recorded by adding a silver nitrate (AgNO_3_) standard solution in small quantities. Then, the chloride-ion content was calculated at the equivalence point using the following equation:

(1) $$Cl\;\left(\%\right)=\frac{3.545\times \left(V_{1}-V_{2}\right)\times 0.05}{W},$$

where *V*_1_ is the volume of the 0.05 N AgNO_3_ solution used for the titration of a sample (mL, equivalence point), *V*_2_ is the volume of the 0.05 N AgNO_3_ solution used for the blank titration of the sample (mL, equivalence point), and *W* is the mass of the sample (g).

### 3.3. Evaluation of Indoor Chloride Attack and Field Exposure Using Fabricated Specimens

#### 3.3.1. Design of Experiments

Table 3 shows the design of experiments for evaluating indoor chloride attack and field exposure using fabricated specimens. Concrete specimens were fabricated using ordinary Portland cement (OPC) and Portland blast furnace slag cement (PBC). The fabricated specimens (OPC) were exposed to pressures of 1 and 6 atm to evaluate the effect of the pressure in the air chamber on chloride-ion penetration. The water-soluble chloride-ion content was measured at various depths and exposure periods. 

**Figure 5:**
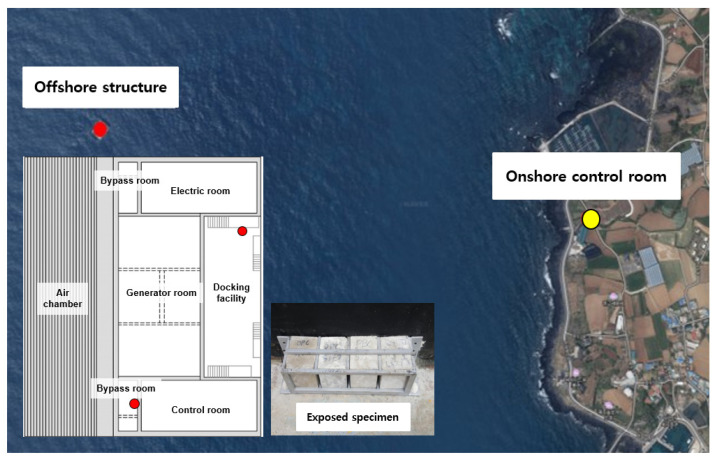
Location of offshore structure (OWC) and onshore control room.

The fabricated specimens were cured for 91 days before the field exposure test to minimize the change in the chloride-ion diffusion coefficient of concrete. The specimen was manufactured with a size of 100 mm × 100 mm × 100 mm, and the location and shape of the field exposure experiment are shown in Figure 5.

The chloride-ion penetration depth and chloride-ion content were measured by exposing the specimens to the following locations: the bypass room, which is subjected to pressure; the docking facility, which is exposed to the marine environment without being affected by pressure; and the onshore control room, where there is a concern for chloride-ion penetration by airborne chlorides [32]. Field exposure was performed for approximately one year in 2018.

#### 3.3.2. Materials

Table 4 lists the physical properties of the materials used in the experiment. The PBC used to fabricate the specimens consisted of 38% PBC, 2.43% SO_3_, and 3.40% MgO. The loss on ignition was 1.71%, corresponding to blast furnace slag cement class 2. The density and specific surface area were 3.05 g/cm^3^ and 4000 cm^2^/g, respectively. The fine and coarse aggregates used in the experiment had densities of 2.56 and 2.67 g/cm^3^, maximum dimensions of 5 and 25 mm, and absorption ratios of 1.01% and 1.39%, respectively. Table 5 shows the composition of the concrete mixture. The water–binder ratio of the mixture was 40%. Additionally, the design compressive strength was set as 35 MPa according to the concrete structure design standard.

#### 3.3.3. Experimental Method

The chloride penetration durability of a marine concrete structure under pressure is less than that of a structure that is not subjected to hydrostatic or air pressure. Hence, to measure the effect of pressure on chloride-ion penetration, a test apparatus was developed to expose the specimens to pressure or chloride ions [33,34].

**Figure 6:**
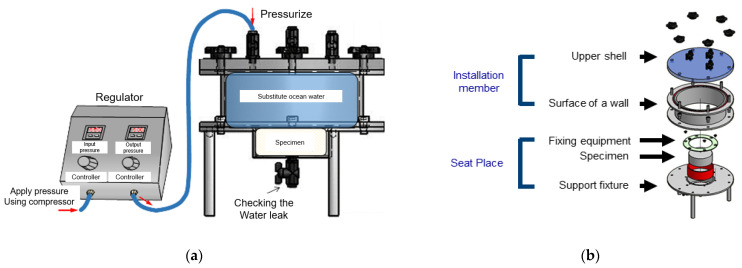
Test apparatus for evaluating chloride-ion penetration in concrete under pressure: (**a**) view map; (**b**) Detail view.

The test apparatus is shown in Figure 6. It is composed of a mounting space for installing a concrete specimen. Specimen fixtures with an inlet and outlet are connected to the mounting space to apply air or water pressure to the specimen. There is a pressurizing part that applies water pressure by supplying seawater to the inlet of the specimen fixture. The central part of concrete specimens is generally used to measure the compressive strength and splitting tensile strength. Thus, the φ 100 mm × 200 mm specimen was cut to a size of φ 100 mm × 50 mm (only using the center 50 mm).

The artificial seawater for the chloride attack environment was produced in accordance with ASTM D1141 [35].

Scanning electron microscopy (SEM) was used to analyze the microstructures of the specimens. The distribution of the pore size and the cumulative pore volume of the specimens were measured using mercury intrusion porosimetry (MIP). The MIP conditions are listed in Table 6.

## 4. Results and Discussion

### 4.1. Chloride-Ion Penetration Behavior through Sampling of Marine Concrete Structure

**Figure 7:**
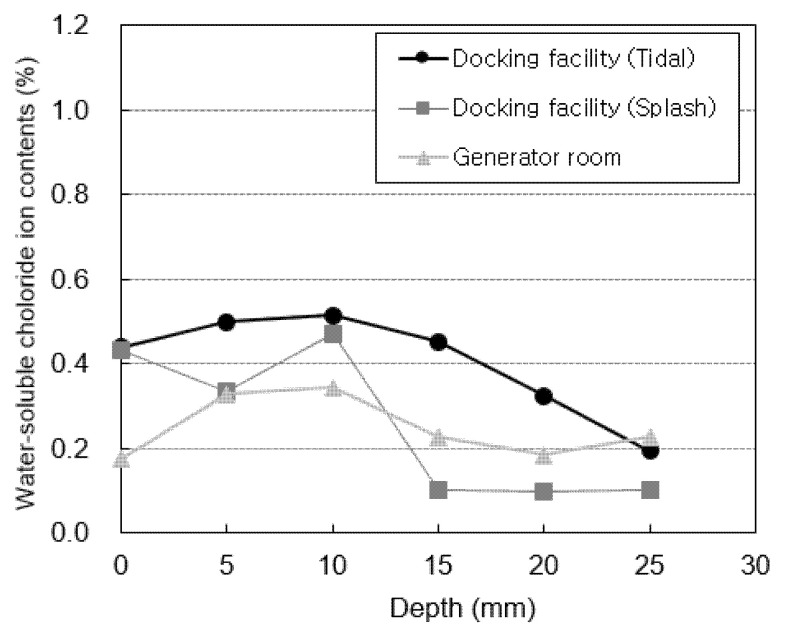
Chloride-ion content at atmospheric pressure.

**Figure 8:**
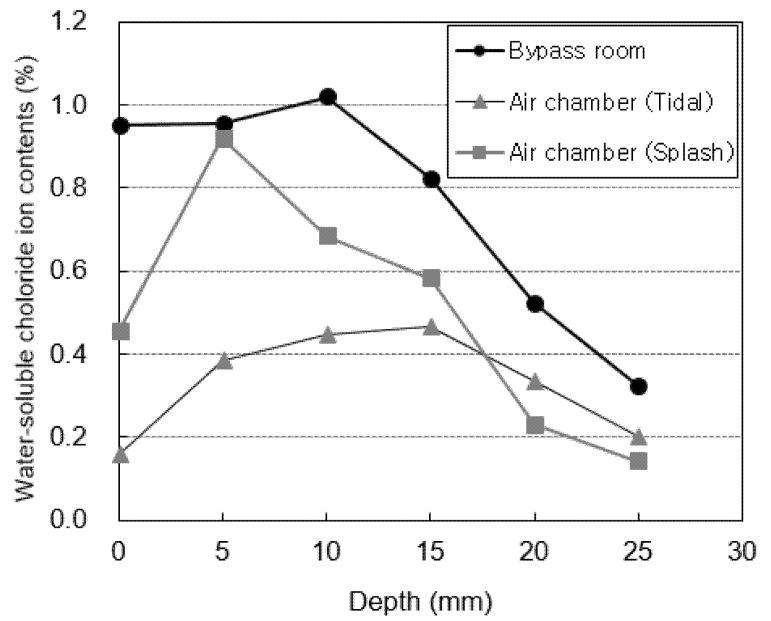
Chloride-ion content under pressure.

Figure 7 shows the water-soluble chloride-ion content measured at locations subjected to atmospheric pressure (the generator room and the tidal and splash zones of the docking facility). Figure 8 shows the water-soluble chloride-ion content measured at locations subjected to pressure (the bypass room and the tidal and splash zones of the air chamber). The highest water-soluble chloride-ion content is measured in the 5–10 mm section for all specimens. After this section, the water-soluble chloride-ion content rapidly decreases as the penetration depth increases. This is considered to be the effect of the diffusion of chloride ions from the surface to the interior. The water-soluble chloride-ion content at a depth of 0 mm, which corresponds to the surface, is relatively low owing to the effect of wind and moisture.

The air chamber and bypass room are repeatedly subjected to positive and negative pressures owing to the nature of the OWC. Thus, the water-soluble chloride-ion content at these locations is higher than that in the docking facility and generator room. In particular, the highest water-soluble chloride-ion content is observed in the bypass room. At the docking facility, the water-soluble chloride-ion content in the tidal zone is higher than that in the splash zone owing to the multi-deterioration resulting from the repeated dry and wet conditions of seawater [36]. However, the opposite trend is observed in the air chamber. This is considered to be because the tidal zone of the air chamber is not affected by pressure because it is immersed in seawater at high tide.

### 4.2. Indoor Chloride Attack Evaluation Using Fabricated Specimens

**Figure 9:**
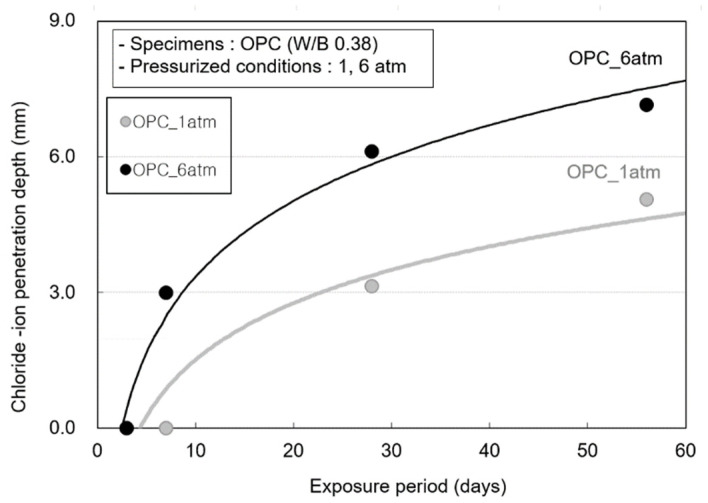
Chloride-ion penetration depth according to the pressure change of the OPC specimen.

Figure 9 shows the chloride-ion penetration depth at various exposure periods. This is because chloride-ion penetration is suppressed by the chloride-ion fixing effect of the blast furnace slag. In the OPC specimens, although chloride-ion penetration does not occur until 3 days, a chloride-ion penetration depth of 2.99 mm is observed at 7 days and 6.12 mm at 28 days.

**Figure 10:**
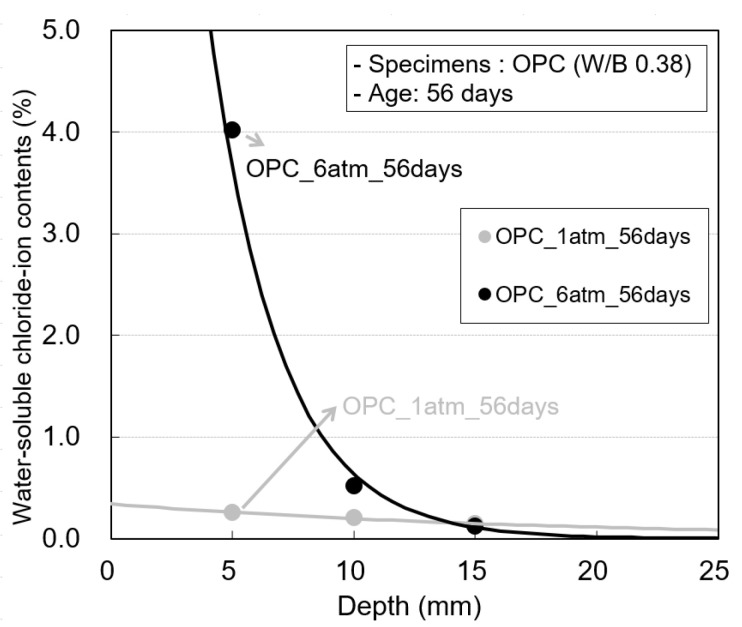
Water-soluble chloride-ion contents according to the pressure change of the OPC specimen.

Figure 10 shows the water-soluble chloride-ion content at hydrostatic pressures of 1 and 6 atm in the OPC specimens at an exposure period of 56 days. The difference between the water-soluble chloride-ion contents at 0–5 mm and 5–10 mm at 6 atm is larger than that at 1 atm. This is because at 6 atm, the chloride-ion content at the concrete surface corresponding to a depth of 0–5 mm increases rapidly owing to sea-water penetration.

### 4.3. Field Exposure Evaluation Using Fabricated Specimens

#### Analysis of Chloride-Ion Content and Chloride-Ion Penetration Depth

**Figure 11:**
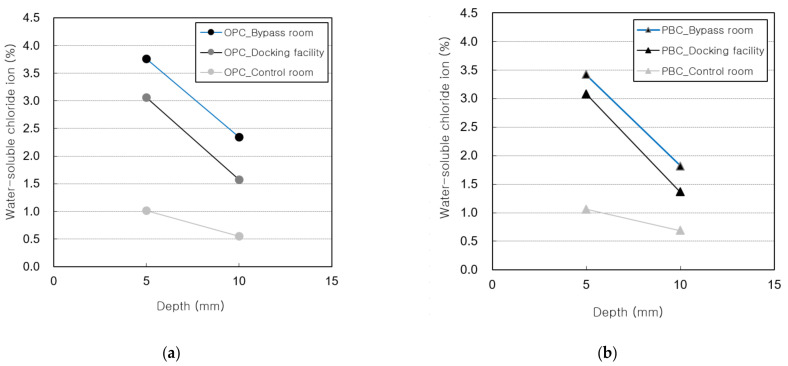
Water-soluble chloride-ion content vs. depth: (**a**) OPC specimens; (**b**) PBC specimens.

**Figure 12:**
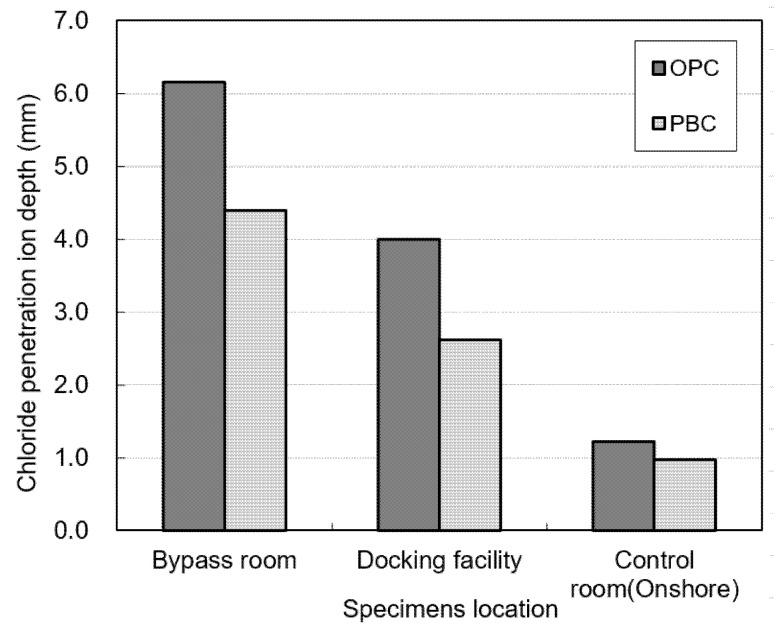
Chloride-ion penetration depth at different exposure locations.

Figure 11 shows the water-soluble chloride-ion contents measured for the OPC and PBC specimens at different exposure locations. The water-soluble chloride-ion content of the OPC specimens is higher than that of the PBC specimens. In addition, the water-soluble chloride-ion content is highest in the bypass room, followed by the docking facility and onshore control room. Figure 12 shows the chloride-ion penetration depth at different exposure locations. The chloride-ion penetration depth in the bypass room is the highest, followed by the docking facility and onshore control room. This trend is similar to the results of the offshore structure sample analysis and experiment with an indoor chloride evaluation test device, which were performed previously.

### 4.4. Changes in Concrete Microstructure According to Pressure

#### 4.4.1. SEM Observation Result

**Figure 13:**
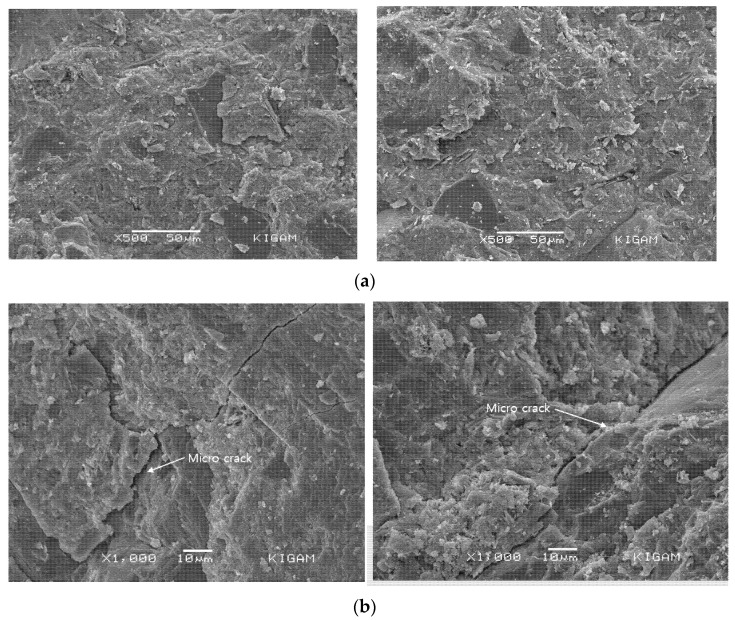
Scanning electron microscopy observation result (by location of exposure): (**a**) atmospheric pressure exposure test specimen (onshore control room); (**b**) air pressure exposure test specimen (bypass room).

Figure 13 shows the SEM observation results for the specimens exposed to the onshore control room and bypass room. A wide-ranging formation of calcium silicate hydrate (C-S-H) and calcium hydroxide (Ca(OH)_2_) is observed in both specimens [37]. The difference in hydration products by cement type is not significant. Furthermore, there are considerably more micro-cracks in the specimen exposed to the bypass room compared to the specimen exposed to the onshore control room [38].

These cracks are considered to occur in the weak part of the concrete matrix owing to complex deteriorations, including the effect of air pressure due to repetitive water level changes.

#### 4.4.2. Changes in Micropore Structure of Concrete

**Figure 14:**
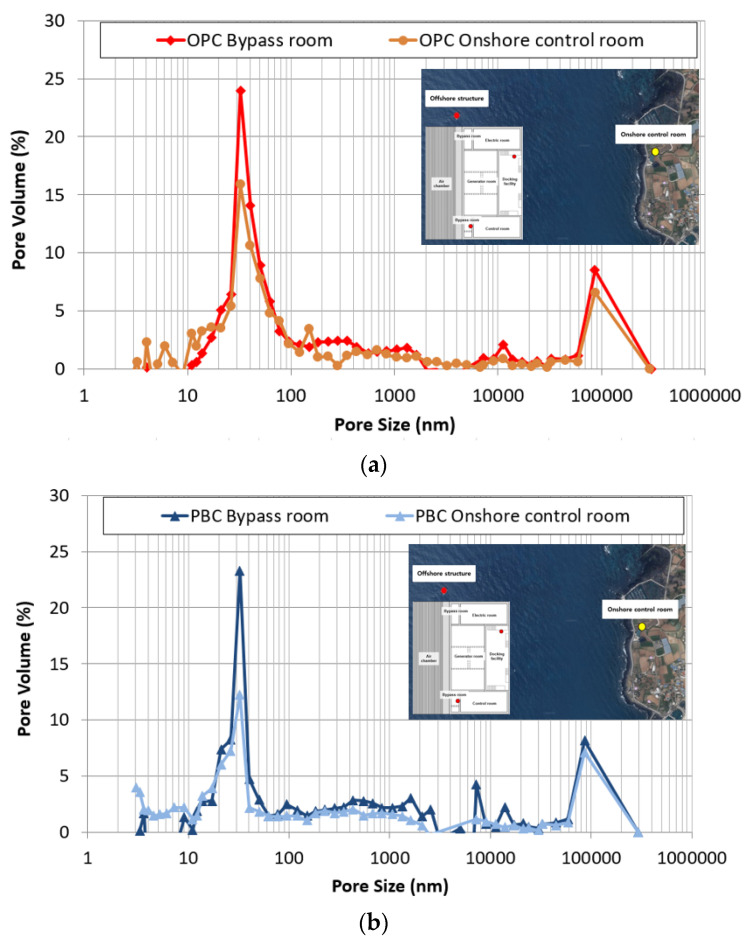
Pore size distribution of concrete at different exposure locations: (**a**) OPC; (**b**) PBC.

Figure 14 shows the distribution of pores by the pore size of the OPC and PBC specimens at different exposure locations. The change in the micropore structure was analyzed to determine the cause of the promotion of chloride penetration due to the previously confirmed pressure. The pores of the concrete are classified into the intermediate, gel, and capillary pores based on the pore size [39]. Among these, capillary pores (5–100 nm) strongly affect the ion penetration of cement hardeners. In the OPC and PBC specimens, capillary pores increase rapidly for specimens exposed to the bypass room. Additionally, capillary pores with a diameter of approximately 50 nm increase significantly. This can be attributed to moisture penetration due to repetitive air pressure. When pressure is applied, the water penetration ratio of concrete increases because of diffusion; this is accompanied by the internal deformation of the concrete. It is estimated that capillary pores with a diameter of 100 nm or lower increase owing to the internal deformation, and the chloride-ion penetration of concrete is accelerated by the expansion of these capillary pores.

### 4.5. Diffusion Coefficient Calculation Using Fick′s Second Law

**Figure 15:**
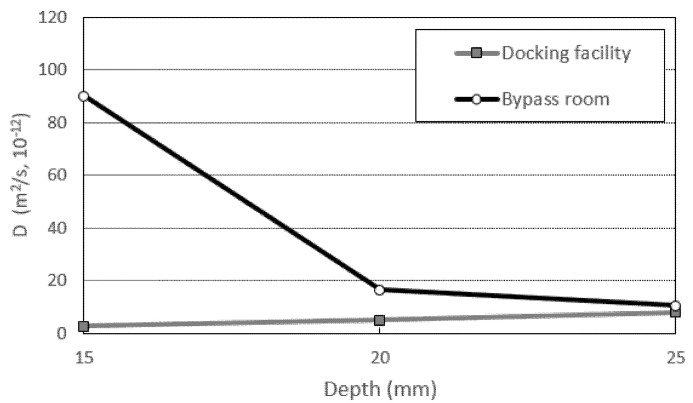
Diffusion coefficient by location (15–25 mm).

Diffusion was analyzed for abnormal states (when the concentration value changes with time) using real sea sampling data. Using the second law, the diffusion coefficient (D) representing the rate of diffusion was calculated as shown in Table 7 and Figure 15.

**Figure 16:**
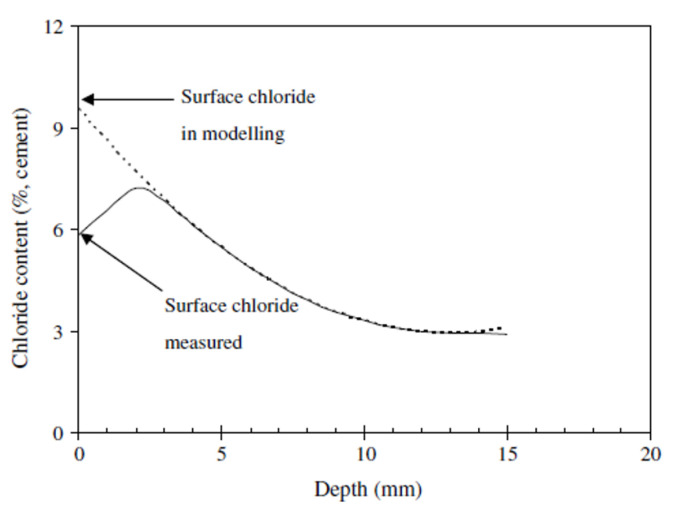
Surface chloride concentration prediction graph (regression) [40].

To calculate the diffusion coefficient (D), the surface chloride concentration value (Cs) should be the maximum value. From the measurements, the maximum chloride concentration value was confirmed at depths of 0 to 10 mm, and there were regions where the chloride concentration was higher on the inside than on the surface; after the maximum amount of chloride, the concentration gradually decreased. Considering these characteristics, the average value of the concentration of chloride at the depths of 0 to 10 mm was used as Cs [40,41]. In general, the Cs calculation is predicted based on ASTM C1556 [42]. As this predicted value is calculated using the regression equation as shown in Figure 16, the amount of surface chloride of 0 mm is calculated as the maximum value. However, from analyzing the measurement data of this study, the maximum chloride concentration value was measured at the depths of 0 to 10 mm. Therefore, using the measurement data is important for calculating the maximum surface chloride concentration. The data was used as the maximum surface chloride concentration by correcting it with the average chloride concentration value of the 0–10 mm measurement data.

Using the corrected maximum Cs, the diffusion coefficients of the docking facility (normal atmospheric pressure) and bypass room (repeated air pressure) were compared. The average diffusion coefficient value of the eyepiece was calculated as 5.214 × 10^−12^ m^2^/s and that of the bypass room as 39 × 10^−12^ m^2^/s. The diffusion coefficient was calculated to be high due to the repeated air pressure during compound deterioration.

It is difficult and dangerous to access marine structures to obtain sea measurement data of offshore concrete structures subjected to repeated air pressure. Therefore, we propose Equation (4) to predict the value of the diffusion coefficient at the location receiving repeated air pressure using the diffusion coefficient value at the location receiving normal atmospheric pressure.

(2) $$\frac{C\left(x,t\right)-C_{0}}{C_{s}-C_{0}}=1-erf\left(\frac{x}{2\sqrt{Dt}}\right),$$

(3) $$D=\frac{1}{t}\times {\left(\frac{x}{2{erf}^{-1}\left(1-\frac{C_{x}}{C_{s}}\right)}\right)}^{2}$$

where

*C*_0_: Initial chloride concentration = 0 (kg/m^3^);
*Cs*: Maximum surface chloride concentration (kg/m^3^);
x: depth (mm);
t: days (Years);
*D*: diffusion coefficient (m^2^/s).
  (4) $$D_{RAP}=D_{GAP}\times 133.01\times 10^{-1.618x}$$
  where
*D_RAP_*: diffusion coefficient at locations subjected to repetitive air pressure;
*D_GAP_*: diffusion coefficient at a location subjected to general atmospheric pressure;
*x*: depth.

## 5. Conclusions

This study analyzed the effects of repetitive air pressures caused by water level changes in wave power marine concrete structures on the chloride-ion diffusion characteristics the structures. The main conclusions were as follows:

1. The diffusion of chloride ions was accelerated by the repetitive application of seawater and air pressures, which acted as deteriorating factors for wave power marine concrete structures owing to the effect of the marine environment. The outer walls of wave power marine concrete structures were subjected to hydrostatic pressure or atmospheric pressure, and the air chamber and bypass room were subjected to the complex effects of repetitive air pressures and high humidity caused by seawater level changes.
2. The water-soluble chloride-ion content was measured by collecting field samples from a marine concrete structure at different locations and performing an exposure test. The air chamber and bypass room, which were under pressure, showed a high water-soluble chloride-ion content, unlike the locations exposed to atmospheric pressure. In the range of 0~15 mm, the amount of chloride was about twice as high in the place where the air pressure was applied. Furthermore, the chloride-ion penetration into concrete under the hydrostatic pressure of seawater was observed using an indoor chloride evaluation device. The water-soluble chloride-ion content rapidly increased with hydrostatic pressure and exposure period.
3. The result of the field exposure test confirmed that chloride-ion diffusion increased due to the repetitive application of air pressure and hydrostatic pressure. In addition, the specimens consisting of blast furnace slag exhibited suppressed chloride-ion penetration. The reason is that the fine powder of blast furnace slag has a dense hardened body and excellent water tightness and durability of abrasion.
4. Micro-cracks were observed in the concrete specimen that was subjected to repetitive air pressures owing to fractures in the weak parts of the concrete matrix. The capillary pores on the concrete surface expanded because of pressure. This suggested that the application of pressure to concrete not only promoted the movement of chloride ions but also chloride penetration via pore expansion.
5. Based on the sea sampling data, the D, which indicates the rate of diffusion, was calculated using Fick′s second law for the location receiving repeated air pressure (bypass room) and general atmospheric pressure (docking facility). The bypass room was confirmed to have a D 7.5 times higher than that of the docking facility. Since it is difficult and dangerous to access the sea for marine concrete structures subjected to repeated air pressure, an equation was proposed to predict the diffusion coefficient value at the location subjected to repeated air pressure using the diffusion coefficient at the location receiving general atmospheric pressure.

## Figures and Tables

**Table 1:** Design of experiments.

Contents	Experimental Plan
Experiment 1	Evaluate chloride-ion penetration behavior by collecting samples from oscillating water column (OWC) -Evaluate the chloride penetration durability of OWC
Experiment 2	Evaluate indoor chloride attack and field exposure using fabricated specimens -Evaluate the effect of pressure on chloride-ion penetration

**Table 2:** Design of experiments 1.

Experimental Variables	Experimental Levels	Evaluation Items
Sampling location	Air chamber, bypass room, generator room, docking facility	Water-soluble chloride-ion content
Sampling depth	0, 5, 10, 15, 20, and 25 (mm)	

**Table 3:** Design of experiments 2.

Classification	Experimental Variables	Experimental Levels	Evaluation Items
Indoor chloride evaluation	Mixing condition (cement type)	Ordinary Portland cement (OPC)	Water-soluble chloride-ion content Chloride-ion penetration depth
	Exposure period	3, 7, 28, and 56 (days)	
	Pressure	1 and 6 (atm)	
Field exposure experiment	Field exposure conditions	Bypass room, docking facility, and onshore control room Fabricated in the same manner as indoor test specimens Exposed (OPC, PBC) from January to December 2018	Chloride-ion penetration depth Chloride-ion content Observation via scanning electron microscopy Pore volume (mercury intrusion porosimetry)

**Table 4:** Physical properties of used materials.

Materials (Sign)	Physical Properties
OPC	Density: 3.12 g/cm^3^, Blaine: 3200 cm^2^/g
PBC	Class 2, Density: 3.05 g/cm^3^, Blaine: 4000 cm^2^/g
Fine aggregate (S)	Density: 2.56 g/cm^3^, Maximum dimensions: 5 mm, Absorption ratio of 1.01%
Coarse aggregate (G)	Density: 2.67 g/cm^3^, Maximum dimensions: 25 mm, Absorption ratio of 1.39%

**Table 5:** Concrete composition.

Specimen	*f_ck_* (MPa)	Slump (mm)	Air Content (%)	W/B (%)	S/a (%)	Unit Weight (kg/m^3^)				
						W	OPC	PBC	S	G
OPC	35	150 ± 20	4.0 ± 1.0	38.2	44.6	164	429	-	752	967
PBC							-	429	748	962

**Table 6:** Mercury intrusion porosimetry conditions.

Property	Value
Contacting angle (°)	130.0000
Mercury density (g/mL)	13.5462
Mercury surface tension (dyn/c)	485.0000
Maximum head pressure (psi)	4.4500
Stem volume (mL)	1.8360
Penetrometer weight (g)	67.1036
Penetrometer volume (mL)	16.4182
Penetrometer constant (/pF)	27.820

**Table 7:** Chloride concentration value and diffusion coefficient by location.

Depth (mm)	Docking Facility (General Atmospheric Pressure)		Bypass Room (Repetitive Air Pressure)	
	Chloride Concentration (%)	Diffusion Coefficient (10^−12^, m^2^/s)	Chloride Concentration (%)	Diffusion Coefficient (10^−12^, m^2^/s)
Cs (0–10 average value)	0.4132	-	0.9746	-
15	0.0966	2.7023	0.8208	90.0327
20	0.1004	5.0077	0.5213	16.4694
25	0.1026	8.0141	0.3233	10.5184

## Data Availability

Not applicable.
